# Reduced Geriatric Nutritional Risk Index Is Associated with Prevalent Diabetes Mellitus and In-Hospital Mortality in Patients Hospitalized with Heart Failure

**DOI:** 10.3390/nu18132198

**Published:** 2026-07-07

**Authors:** Constanta Corina Nitu, Victoria Ancuta Nyulas, Florina Ruta, Tiberiu Nyulas, Sara Suciu, Ionel Nitu, Florentina Simona Toncean, Septimiu Voidăzan

**Affiliations:** 1 Doctoral School, George Emil Palade University of Medicine, Pharmacy, Science and Technology of Targu Mures, Gheorghe Marinescu Street No. 38, 540139 Targu Mures, Romania; nitucorina31@gmail.com (C.C.N.); av_nitu@hotmail.com (I.N.); 2 Department of Medical Informatics and Biostatistics, George Emil Palade University of Medicine, Pharmacy, Science and Technology of Targu Mures, Gheorghe Marinescu Street No. 38, 540136 Targu Mures, Romania; 3 Department of Community Nutrition and Food Safety, George Emil Palade University of Medicine, Pharmacy, Science and Technology of Targu Mures, Gheorghe Marinescu Street No. 38, 540136 Targu Mures, Romania; 4 Department of Physiology, George Emil Palade University of Medicine, Pharmacy, Science and Technology of Targu Mures, Gheorghe Marinescu Street No. 38, 540136 Targu Mures, Romania; tiberiu.nyulas@umfst.ro; 5 Faculty of General Medicine, George Emil Palade University of Medicine, Pharmacy, Science and Technology of Targu Mures, Gheorghe Marinescu Street No. 38, 540136 Targu Mures, Romania; suciusara@yahoo.com; 6 Department of Management, Dr. Eugen Nicoară Municipal Hospital, 545300 Reghin, Romania; s.boariu@yahoo.com; 7 Department of Epidemiology, George Emil Palade University of Medicine, Pharmacy, Science and Technology of Targu Mures, Gheorghe Marinescu Street No. 38, 540136 Targu Mures, Romania; septimiu.voidazan@umfst.ro

**Keywords:** heart failure, nutritional status, malnutrition, geriatric nutritional risk index

## Abstract

**Background:** Malnutrition is increasingly recognized as an important determinant of prognosis in patients with heart failure (HF). The Geriatric Nutritional Risk Index (GNRI) is a simple nutritional assessment tool associated with adverse cardiovascular outcomes, but its relationship with diabetes mellitus and in-hospital mortality in hospitalized HF patients remains incompletely characterized. This study evaluated the association between GNRI-defined nutritional risk, diabetes mellitus, and in-hospital mortality in patients hospitalized with HF. **Methods:** This prospective observational study included 278 consecutive patients hospitalized with HF at a tertiary cardiology center between September 2025 and March 2026. Nutritional status was assessed using GNRI, and patients were classified as having no nutritional risk (GNRI ≥ 98) or GNRI-defined nutritional risk (GNRI < 98). Logistic regression evaluated the association between GNRI and diabetes mellitus, while Cox proportional hazards regression and Kaplan–Meier analyses assessed in-hospital mortality. **Results:** GNRI-defined nutritional risk was identified in 42.1% of patients. Compared with patients without nutritional risk, those with GNRI-defined nutritional risk had a higher prevalence of diabetes mellitus (73.5% vs. 50.9%, *p* < 0.001), longer hospital stays, and a nearly threefold higher in-hospital mortality rate (29.1% vs. 10.6%, *p* < 0.001). Higher GNRI values were associated with a lower prevalence of diabetes mellitus. Lower GNRI values were also associated with higher in-hospital mortality in the main Cox models; however, this association was attenuated and no longer statistically significant after adjustment for markers of heart failure severity. **Conclusions:** Lower GNRI values identified patients with greater nutritional and clinical vulnerability during hospitalization for HF. Further studies are needed to determine whether GNRI provides incremental prognostic value beyond established HF risk markers.

## 1. Introduction

Heart failure (HF) remains one of the leading causes of morbidity and mortality worldwide and represents a major public health challenge [1,2,3]. Despite significant advances in pharmacological therapies and device-based treatments, the prognosis of patients with HF remains poor, particularly among elderly individuals with multiple comorbidities [3,4]. The global prevalence of HF has increased in recent decades, mainly due to population aging and improved survival following acute cardiovascular events [1,2,5]. In this context, identifying additional clinical factors associated with prognosis has become an important priority in cardiovascular research.

Among these factors, nutritional status has received increasing attention because malnutrition is frequently encountered in patients with HF. Depending on the population studied and the nutritional assessment method applied, its prevalence ranges from approximately 20% to 50%, and it has been consistently associated with higher mortality rates, longer hospital stays, and an increased risk of rehospitalization [6,7]. The mechanisms underlying malnutrition in HF are multifactorial and include systemic inflammation, neurohormonal activation, reduced appetite, gastrointestinal congestion, impaired nutrient absorption, and a persistent catabolic state. Together, these processes contribute to the progressive loss of muscle mass and nutritional reserves, ultimately leading to sarcopenia, frailty, and worsening clinical status [8,9,10].

Given the prognostic importance of nutritional status in HF, several screening tools have been developed to identify patients at nutritional risk. Among them, the Geriatric Nutritional Risk Index (GNRI) has gained considerable attention because it is simple, objective, and easy to use in routine clinical practice. Based on serum albumin levels and the relationship between actual and ideal body weight, GNRI has been shown to provide valuable prognostic information, with lower scores consistently associated with adverse cardiovascular outcomes and poorer survival in patients with cardiovascular disease [11,12,13,14].

In addition to its association with adverse clinical outcomes, nutritional status may also be related to metabolic disorders frequently observed in patients with HF, particularly diabetes mellitus. Diabetes is one of the most common comorbidities in HF and contributes substantially to disease progression and unfavorable outcomes. The coexistence of HF and diabetes is associated with higher hospitalization rates, increased healthcare costs, and reduced survival [15]. Recent studies [16,17,18] have shown that nutritional risk indices, including GNRI and CONUT, are associated not only with cardiovascular outcomes but also with metabolic disturbances. Nutritional imbalance has been linked to insulin resistance, systemic inflammation, and alterations in endocrine regulation, mechanisms commonly observed in patients with diabetes mellitus [19,20]. Therefore, evaluating the relationship between nutritional status and diabetes may provide additional insights into the complex metabolic interactions observed in patients with HF [21,22].

Despite growing evidence supporting the prognostic importance of nutritional status in cardiovascular disease, the interplay between malnutrition, diabetes mellitus, and clinical outcomes in patients hospitalized with HF remains insufficiently characterized [23,24,25]. Both nutritional impairment and diabetes are highly prevalent in this population and have independently been associated with adverse outcomes; however, it remains unclear whether their coexistence identifies a subgroup of patients with greater clinical vulnerability and poorer short-term prognosis. Because nutritional status is not routinely incorporated into traditional cardiovascular risk assessment, a better understanding of its relationship with diabetes and mortality may provide complementary information for risk stratification during hospitalization. Given that GNRI is simple, objective, and based on routinely available clinical parameters, clarifying its clinical relevance in this setting may help improve the overall assessment of patients hospitalized with HF.

The aim of this study was to evaluate the association between nutritional status, as determined by GNRI, and the presence of diabetes mellitus in patients hospitalized with heart failure, as well as to investigate the relationship between GNRI and in-hospital mortality. In addition, the study aimed to perform subgroup analyses to determine whether these associations differed according to relevant clinical characteristics, laboratory parameters, and cardiovascular comorbidities.

## 2. Materials and Methods

### 2.1. Experimental Approach to the Problem

This prospective observational study included consecutively hospitalized patients with acute decompensated heart failure admitted to the Cardiology Department of a tertiary care hospital in Târgu Mureș, Romania, between September 2025 and March 2026. The study was designed to investigate the association between nutritional status, assessed using the Geriatric Nutritional Risk Index (GNRI), diabetes mellitus, and in-hospital mortality in patients hospitalized with heart failure. Clinical, demographic, laboratory, echocardiographic, and therapeutic data were prospectively collected during hospitalization and analyzed to explore the relationship between nutritional status and adverse clinical outcomes.

The study was conducted in accordance with the principles of the Declaration of Helsinki and was approved by the institutional ethics committee of the participating hospital (Decision No. 21410/10 September 2025). Written informed consent was obtained from all participants prior to study inclusion.

### 2.2. Participants

A total of 278 consecutively admitted patients were included in the study. Eligible participants were adults aged 18 years or older with a confirmed diagnosis of heart failure according to current European Society of Cardiology (ESC) guideline criteria. The diagnosis was established based on compatible clinical symptoms and signs together with objective evidence of structural and/or functional cardiac abnormalities documented by transthoracic echocardiography [26]. Patients were subsequently classified as having heart failure with preserved ejection fraction (HFpEF), mildly reduced ejection fraction (HFmrEF), or reduced ejection fraction (HFrEF) according to current ESC recommendations.

Patients with incomplete clinical or laboratory data required for GNRI calculation were excluded. In addition, patients with active malignancies, severe infections requiring intensive care management, or other conditions that could substantially affect inflammatory or nutritional status were not included in the final analysis.

### 2.3. Clinical, Anthropometric and Nutritional Assessment

Clinical, demographic, laboratory, anthropometric, echocardiographic, and therapeutic data were extracted from electronic medical records and hospital databases by members of the research team using a standardized data collection form. Information related to hospitalization, including the type of admission (emergency or elective), length of hospital stay, and previous hospitalizations, was also recorded. All variables were verified for completeness before inclusion in the final database.

Anthropometric assessment was performed at admission or within the first 24 h of hospitalization, before nutritional status classification. Body weight was measured using calibrated hospital scales, with patients wearing light clothing and no shoes whenever their clinical condition allowed. In patients unable to stand safely because of acute decompensated heart failure or severe functional limitation, body weight recorded during routine admission assessment was used. Height was measured using a standard stadiometer or obtained from recent medical records when direct measurement was not feasible. Body mass index (BMI) was calculated as weight in kilograms divided by height in meters squared, and the presence of abdominal obesity was also documented. To reduce measurement error, anthropometric data were verified against previous medical records whenever available, and incomplete or inconsistent measurements were excluded from the analysis.

Cardiovascular assessment included heart failure type, New York Heart Association (NYHA) functional class, left ventricular ejection fraction (LVEF), atrial fibrillation, coronary artery disease, angina pectoris, previous myocardial infarction, ischemic stroke, and arterial hypertension. Associated comorbidities, including diabetes mellitus, anemia, dyslipidemia, and chronic kidney disease, were also recorded. Diabetes mellitus was defined based on a documented medical history of diabetes recorded in the electronic medical record, current use of glucose-lowering medication, or a previously established diagnosis confirmed by the treating physician.

Information regarding alcohol consumption and smoking status was obtained from admission interviews and electronic medical records as part of routine clinical assessment. Both variables were recorded based on patient self-report and categorized as present or absent.

Laboratory parameters obtained at admission included hemoglobin, hematocrit, red blood cell count, serum albumin, serum iron, ferritin, blood glucose, creatinine, estimated glomerular filtration rate (eGFR), serum sodium and potassium, NT-proBNP, liver enzymes (AST and ALT), and lipid profile. Serum albumin concentrations were measured at admission or within the first 24 h of hospitalization as part of routine clinical laboratory testing and were subsequently used for GNRI calculation and nutritional risk classification.

Echocardiographic assessment was performed using transthoracic echocardiography according to standard clinical practice protocols. Left ventricular ejection fraction was calculated using the biplane Simpson method. Echocardiographic evaluation also included the assessment of cardiac chamber dimensions, wall motion abnormalities, valvular disease, and other relevant structural cardiac findings when available. Based on LVEF values, patients were classified as having preserved ejection fraction (≥50%), mildly reduced ejection fraction (40–49%), or reduced ejection fraction (<40%), in accordance with current guideline recommendations and previous cardiovascular studies evaluating nutritional status in heart failure populations [27,28,29].

### 2.4. GNRI Calculation and Nutritional Classification

Nutritional status was assessed using the Geriatric Nutritional Risk Index (GNRI), calculated according to the original formula proposed by Bouillanne et al. [30]:

GNRI = (1.489 × serum albumin [g/L]) + (41.7 × body weight/ideal body weight)

Although serum albumin values are reported in the tables as g/dL, values used for GNRI calculations were converted to g/L before applying the formula. In accordance with the original GNRI methodology, the body weight-to-ideal body weight ratio was set to 1 when actual body weight exceeded ideal body weight. Ideal body weight was calculated using the Lorentz formula.

GNRI was selected because it is an objective, reproducible, and easily applicable nutritional risk index based on routinely available clinical parameters. Previous studies have validated its use in elderly, hospitalized, and cardiovascular populations, demonstrating associations with functional decline, prolonged hospitalization, adverse clinical outcomes, and mortality. In patients with heart failure, GNRI has been widely used as a practical tool for nutritional risk assessment and prognostic evaluation. However, it should be interpreted as an indicator of nutritional risk rather than a comprehensive diagnostic tool for malnutrition.

According to previously established cutoff values, patients were classified into two categories: GNRI ≥ 98 (no nutritional risk) and GNRI < 98 (presence of GNRI-defined nutritional risk) [28,30,31,32].

For comparative purposes, the Controlling Nutritional Status (CONUT) score was also calculated using routinely available laboratory parameters, as it has been widely applied for nutritional assessment in patients with cardiovascular disease [30].

### 2.5. Statistical Analysis

The primary analyses evaluated the association between GNRI and diabetes mellitus, as well as the relationship between GNRI and in-hospital mortality. Additional analyses explored the association between nutritional status and relevant clinical characteristics.

Continuous variables were expressed as mean ± standard deviation (SD) or median with interquartile range (IQR), according to data distribution. Categorical variables were presented as frequencies and percentages. Patients with incomplete clinical or laboratory information required for GNRI calculation were excluded before inclusion in the final study cohort. A limited number of missing values remained for some laboratory, echocardiographic, and treatment-related variables because these data were not available for all patients in the electronic medical records. No missing-data imputation procedures were applied, and all analyses were performed using the available data for each variable (complete-case analysis). Consequently, the number of observations may vary slightly across individual variables and statistical analyses.

Comparisons between groups were performed using Student’s *t*-test or the Mann–Whitney U test for continuous variables and the chi-square test or Fisher’s exact test for categorical variables, as appropriate.

The association between GNRI and diabetes mellitus was evaluated using binary logistic regression models, and results were reported as odds ratios (ORs) with 95% confidence intervals (CIs). Model 1 was unadjusted, Model 2 was adjusted for age and sex, and Model 3 was additionally adjusted for body mass index, abdominal obesity, smoking status, alcohol consumption, coronary artery disease, angina pectoris, previous myocardial infarction, ischemic stroke, and hypertension.

The relationship between GNRI and in-hospital mortality was assessed using Cox proportional hazards regression models, with hazard ratios (HRs) and 95% confidence intervals reported. Model 1 was unadjusted, Model 2 was adjusted for age and sex, Model 3 was additionally adjusted for body mass index, abdominal obesity, smoking status, and alcohol consumption, and Model 4 was further adjusted for coronary artery disease, angina pectoris, previous myocardial infarction, ischemic stroke, and hypertension.

Given the limited number of in-hospital deaths, a sensitivity analysis using a more parsimonious model was also performed to reduce the risk of overfitting. This model included clinically pre-specified covariates considered most relevant for in-hospital mortality: age, sex, body mass index, diabetes mellitus, and coronary heart disease.

Covariates included in multivariable models were selected a priori based on clinical relevance and evidence from previous studies reporting their association with nutritional status, diabetes mellitus, and adverse outcomes in heart failure. Multicollinearity among covariates was assessed before model construction, and no clinically relevant collinearity was identified. For Cox proportional hazards analyses, the proportional hazards assumption was evaluated using graphical inspection of log-minus-log survival plots and was considered acceptable.

Subgroup analyses were performed to determine whether the association between GNRI and diabetes mellitus differed according to sex, age, obesity status, smoking status, alcohol consumption, cardiovascular comorbidities, and hospitalization characteristics. Interaction terms were included in regression models to formally test for effect modification across subgroups.

Kaplan–Meier survival curves and log-rank tests were used to compare in-hospital survival according to GNRI-defined nutritional status. For survival analyses, the time origin was defined as the date of hospital admission. Follow-up time corresponded to the duration of hospitalization, measured from admission until either in-hospital death or discharge. Patients discharged alive were censored at the time of discharge.

All statistical analyses were performed using IBM SPSS Statistics for Windows, version 26.0 (IBM Corp., Armonk, NY, USA), and a two-sided *p*-value < 0.05 was considered statistically significant.

## 3. Results

**Figure 1:**
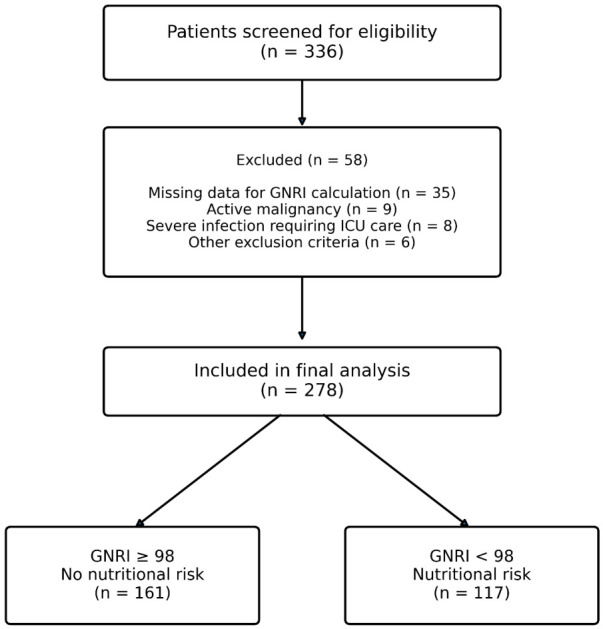
Selection and classification of hospitalized patients with heart failure according to nutritional status assessed using the Geriatric Nutritional Risk Index (GNRI). Patients were categorized into two groups: GNRI ≥ 98 (no nutritional risk) and GNRI < 98 (GNRI-defined nutritional risk). Subsequent analyses included logistic regression to evaluate the association between GNRI and diabetes mellitus and Cox proportional hazards regression with Kaplan–Meier survival analysis to assess the relationship between GNRI and in-hospital mortality.

A total of 336 patients were screened during the study period. After applying the predefined exclusion criteria, 278 patients were included in the final analysis. Specifically, 35 patients were excluded because of incomplete data required for GNRI calculation, 9 because of active malignancy, 8 because of severe infection requiring intensive care management, and 6 because of other exclusion criteria (Figure 1).

### 3.1. Personal, Demographic, and Behavioral Data

The study cohort included 278 patients, of whom 161 were classified in the group without GNRI-defined nutritional risk (GNRI ≥ 98) and 117 in the GNRI-defined nutritional risk group (GNRI < 98).

Patients with GNRI-defined nutritional risk experienced a less favorable clinical course, characterized by a nearly threefold higher in-hospital mortality rate and longer hospitalization. Smoking and alcohol consumption were also more frequent in this group, whereas demographic characteristics and anthropometric measures were generally comparable between groups (Table 1). The median duration of hospitalization was approximately three days longer in patients with GNRI-defined nutritional risk.

### 3.2. Nutritional Status and Biochemical Parameters

Patients with GNRI-defined nutritional risk exhibited a less favorable hematological, nutritional, and biochemical profile, characterized by lower albumin concentrations, higher CONUT scores, more frequent anemia, and multiple laboratory abnormalities consistent with impaired nutritional status (Table 1). Detailed laboratory and biochemical characteristics are presented in Supplementary Table S1.

### 3.3. Cardiovascular Pathology

Cardiovascular characteristics are summarized in Table 2. Patients with GNRI-defined nutritional risk more frequently presented coronary artery disease, angina pectoris, previous myocardial infarction, conduction disorders, and reduced left ventricular ejection fraction. No significant differences were observed regarding atrial fibrillation or electrocardiographic parameters.

### 3.4. Associated Pathologies

Patients with GNRI-defined nutritional risk exhibited a less favorable comorbidity profile, characterized by an approximately 45% higher prevalence of diabetes mellitus and markedly higher frequencies of anemia, thromboembolic events, infectious complications, and diabetes-related conditions (Table 2). The magnitude of this difference suggests that poorer nutritional status identifies a subgroup with substantial metabolic vulnerability. Additional comorbidities and treatment characteristics are presented in Supplementary Tables S2 and S3.

### 3.5. Medication

Most pharmacological treatments were similarly distributed between groups. The only significant difference was observed for insulin therapy, which was more frequently used among patients with GNRI-defined nutritional risk (Table 3).

### 3.6. Association Between GNRI-Defined Nutritional Risk, Diabetes Mellitus, and In-Hospital Mortality in Patients with Heart Failure

Higher GNRI values were independently associated with a lower prevalence of diabetes mellitus across all regression models. Quartile analyses demonstrated a dose–response relationship, with progressively higher odds of diabetes observed across decreasing GNRI quartiles. Similar findings were observed when GNRI was analyzed as a categorical variable using the predefined GNRI cutoff (Table 4).

**Figure 2:**
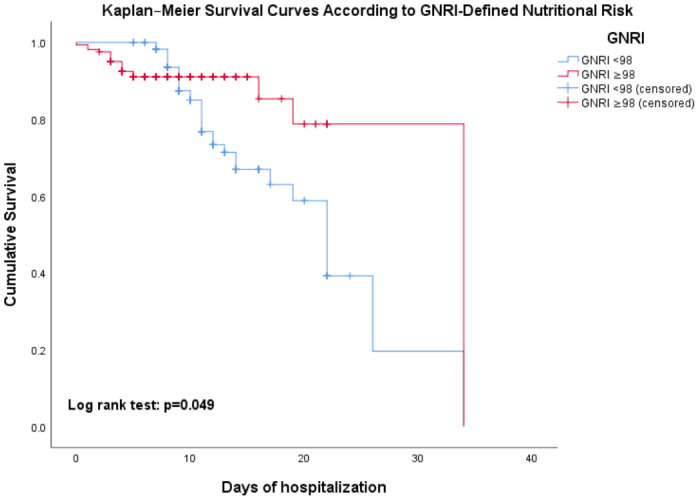
Kaplan–Meier curves for in-hospital survival according to GNRI-defined nutritional risk.

Kaplan–Meier curves suggested lower survival among patients with GNRI-defined nutritional risk, with a borderline significant difference between groups (log-rank *p* = 0.049) (Figure 2).

Cox proportional hazards regression analysis was performed to evaluate the association between GNRI and mortality in patients with heart failure.

Lower GNRI values were generally associated with a higher risk of in-hospital mortality across the Cox regression models, although these findings should be interpreted cautiously given the limited number of events and the possibility of residual confounding. In the fully adjusted model, patients in the lowest GNRI quartile had an approximately 2.7-fold higher mortality risk compared with those in the highest quartile (Table 5). The magnitude of this association suggests that poorer nutritional status identifies a subgroup with substantially greater short-term clinical vulnerability during hospitalization.

To further evaluate the robustness of the findings, we also assessed the association between GNRI and in-hospital mortality using multivariable logistic regression. The results were broadly consistent with those obtained in the Cox models and are presented in Supplementary Table S4.

Sensitivity analyses using more parsimonious Cox regression models yielded similar findings, although statistical significance became borderline in the most adjusted model (Supplementary Table S5).

Finally, in a sensitivity analysis additionally adjusted for markers of heart failure severity, including left ventricular ejection fraction, New York Heart Association functional class, ln(NT-proBNP), and estimated glomerular filtration rate, the association between GNRI and in-hospital mortality was attenuated and no longer remained statistically significant (Supplementary Table S6).

Taken together, these findings suggest that poorer nutritional status, reflected by lower GNRI values, is associated with a higher prevalence of diabetes mellitus and identifies a subgroup of patients with higher crude in-hospital mortality and greater clinical vulnerability. However, after additional adjustment for markers of heart failure severity, the association between GNRI and in-hospital mortality was attenuated and no longer statistically significant.

Subgroup analyses were performed to evaluate whether the association between GNRI and diabetes mellitus differed according to relevant clinical characteristics (Table 6). These analyses were exploratory in nature. Overall, the association between GNRI and diabetes mellitus remained generally consistent across the analyzed subgroups, including categories defined by sex, age, obesity status, smoking status, alcohol consumption, cardiovascular comorbidities, anemia, and dyslipidemia. Although the association appeared stronger among patients with abdominal obesity, only the interaction term for abdominal obesity reached statistical significance (*p* for interaction = 0.04). No significant interactions were observed for the other predefined subgroup variables.

These findings suggest that the association between lower GNRI values and a higher prevalence of diabetes mellitus remains relatively stable across a broad range of clinical characteristics, while the presence of abdominal obesity may influence the strength of this relationship (Table 6).

## 4. Discussion

Our findings also have potential clinical implications regarding the relationship between nutritional status and diabetes mellitus in patients with heart failure. Since diabetes mellitus was a pre-existing diagnosis in our study population, the observed association should be interpreted as a relationship between lower GNRI values and a higher prevalence of diabetes rather than as evidence that nutritional risk contributes to the development of diabetes. These findings suggest that patients with heart failure and diabetes are more likely to present concomitant nutritional impairment, identifying a subgroup characterized by greater clinical vulnerability. Although diabetes is routinely included in cardiovascular risk assessment, it does not provide information regarding nutritional reserves or protein-energy status. In this context, GNRI may offer complementary information beyond traditional risk factors and may help identify patients who could benefit from closer nutritional monitoring and individualized multidisciplinary management during hospitalization.

The observed association between GNRI-defined nutritional risk and increased in-hospital mortality suggests that poorer nutritional status identifies a subgroup of patients with heart failure who experience a less favorable short-term clinical course during hospitalization. However, in an additional sensitivity analysis adjusted for markers of heart failure severity, including left ventricular ejection fraction, New York Heart Association functional class, ln(NT-proBNP), and estimated glomerular filtration rate, this association was attenuated and no longer remained statistically significant. These findings suggest that lower GNRI values may primarily identify patients with a greater burden of cardiac dysfunction and overall clinical vulnerability rather than acting as an independent prognostic marker beyond established indicators of heart failure severity.

Patients with diabetes frequently present insulin resistance, altered body composition, oxidative stress, and chronic low-grade inflammation, factors that may adversely affect nutritional status and contribute to overall clinical vulnerability. These mechanisms may partly explain the higher prevalence of diabetes among patients with GNRI-defined nutritional risk in our cohort.

An alternative explanation should also be considered. Patients with long-standing diabetes mellitus often have a greater burden of cardiovascular comorbidities, chronic inflammation, microvascular and macrovascular complications, and reduced functional capacity, factors that may negatively affect nutritional status. Therefore, the higher prevalence of GNRI-defined nutritional risk observed in diabetic patients may reflect the greater overall disease burden associated with diabetes rather than a direct relationship between nutritional risk and diabetes itself. Given the observational design of the present study, the direction of this association cannot be established, and the findings should be interpreted as demonstrating an association between coexisting conditions rather than a causal relationship.

### 4.1. Nutritional Risk in Patients with Heart Failure

Malnutrition is increasingly recognized as a frequent condition among patients with heart failure, especially in elderly individuals with multiple comorbidities. The prevalence of malnutrition in heart failure varies considerably across studies and has been estimated to range from approximately 20% to 60%, depending on the assessment method used [33,34,35].

In the present study, patients classified as having GNRI-defined nutritional risk exhibited significant biochemical abnormalities, including lower levels of albumin, hemoglobin, hematocrit, iron-related micronutrient parameters, and electrolytes. These findings are consistent with previous studies showing that malnutrition in cardiovascular patients is frequently accompanied by anemia, micronutrient deficiencies [36], iron deficiency, and systemic metabolic disturbances.

Previous investigations conducted in cardiovascular populations have reported similar associations. Rus et al. demonstrated that impaired nutritional status in patients with acute myocardial infarction is associated with increased systemic vulnerability and activation of inflammatory processes [37,38]. Similarly, Czinege et al. reported that deterioration of nutritional status is closely linked to reduced left ventricular ejection fraction and the occurrence of adverse cardiovascular outcomes following myocardial infarction [39,40].

An important observation in our study was the absence of significant differences in anthropometric parameters, such as body mass index, between patients with and without malnutrition. This finding highlights the well-known limitations of BMI as a marker of nutritional status in chronic diseases. In cardiovascular patients, malnutrition often reflects a qualitative protein deficit rather than simple weight loss. This condition may progress toward cardiac cachexia, a severe and frequently underrecognized complication associated with poor prognosis in patients with heart failure [41].

This phenomenon, often referred to as “hidden malnutrition,” has been described in numerous studies investigating nutritional risk in cardiovascular populations.

### 4.2. GNRI and Metabolic Dysregulation

One of the most important findings of our study was the strong association between GNRI and diabetes mellitus. Logistic regression analyses demonstrated that higher GNRI values were associated with a lower prevalence of diabetes mellitus, even after adjustment for potentially confounding demographic and metabolic factors.

Furthermore, the quartile-based analysis revealed a clear dose–response relationship, with lower GNRI quartiles being associated with a progressively higher prevalence of diabetes mellitus. Patients in the lowest GNRI quartile had more than twice the prevalence of diabetes mellitus compared with those in the highest quartile.

From a clinical perspective, the approximately 45% higher prevalence of diabetes and the more than twofold higher odds of diabetes observed in the lowest GNRI quartile suggest that poorer nutritional status identifies a subgroup with substantial metabolic vulnerability.

The relationship between nutritional status and metabolic disorders may appear paradoxical, especially considering the traditional association between diabetes and obesity. However, increasing evidence suggests that malnutrition and metabolic dysregulation frequently coexist in patients with chronic cardiovascular diseases. Recent research has highlighted the role of nutritional imbalance in the development of metabolic dysfunction. Dina et al. emphasized that dietary patterns and metabolic disturbances contribute to cardiovascular disease development through multiple molecular mechanisms, including oxidative stress, chronic inflammation, and endocrine dysregulation [42]. These mechanisms are also involved in the pathogenesis of insulin resistance and diabetes mellitus.

Similarly, studies investigating nutritional risk scores in hospitalized cardiovascular patients have demonstrated strong associations between malnutrition and metabolic disorders [29,43]. In a prospective study evaluating the CONUT score in hospitalized cardiovascular patients, Dina et al. reported that malnutrition was associated with multiple clinical and metabolic abnormalities, including impaired glucose metabolism and increased disease complexity [29].

Another investigation focusing on GNRI-defined malnutrition in hospitalized cardiovascular patients demonstrated significant associations between nutritional risk, biomarker profiles, and treatment patterns [44].

Taken together, these findings suggest that nutritional status may represent an important marker of metabolic vulnerability in patients with cardiovascular diseases.

### 4.3. GNRI and Clinical Outcomes

In addition to its association with diabetes mellitus, our study showed that lower GNRI values were associated with in-hospital mortality. This association persisted across the main multivariable models but should be interpreted cautiously given the limited number of events, the possibility of residual confounding, and the absence of several established markers of heart failure severity from the adjusted analyses. Therefore, although lower GNRI values remained associated with adverse in-hospital outcomes, the present findings should be considered hypothesis-generating and require confirmation in larger studies. These observations support the concept that nutritional risk reflects more than nutritional status alone and may represent a broader marker of overall physiological reserve and clinical vulnerability in patients with heart failure. A lower GNRI score may capture the combined effects of inflammation, catabolic activation, impaired functional reserve, and metabolic disturbances, all of which are known to contribute to adverse clinical outcomes in this population.

One of the most clinically relevant findings of the present study was the nearly threefold higher in-hospital mortality observed among patients with GNRI-defined nutritional risk compared with those without nutritional risk.

Interestingly, NT-proBNP levels did not differ significantly between patients with and without nutritional risk, despite the higher mortality observed among patients with lower GNRI values. Although the reasons for this observation cannot be determined from the present study, it may suggest that GNRI and NT-proBNP are associated with different dimensions of clinical status in patients hospitalized with heart failure. This finding should be interpreted cautiously, particularly because the association between GNRI and in-hospital mortality was no longer statistically significant after adjustment for heart failure severity markers, including ln(NT-proBNP).

These findings are consistent with previous studies investigating the prognostic value of GNRI in heart failure. The study conducted by Ono et al. demonstrated that moderate-to-severe nutritional risk defined by GNRI is associated with significantly increased mortality following hospitalization for acute heart failure [44].

The association between GNRI and mortality is likely multifactorial. Because GNRI incorporates both serum albumin concentration and the relationship between actual and ideal body weight, it may reflect not only nutritional depletion but also the presence of chronic inflammation, disease severity, and reduced physiological reserve. This broader biological significance may explain why GNRI has consistently demonstrated prognostic value across different cardiovascular populations.

Previous studies have reported that nutritional risk assessed using the CONUT score is significantly associated with both diabetes mellitus and increased mortality in patients with heart failure [45]. In line with these findings, our results indicate that GNRI-defined nutritional risk is also associated with a higher prevalence of diabetes and an increased risk of in-hospital mortality.

The mechanisms linking malnutrition to adverse cardiovascular outcomes are complex and multifactorial. Protein-energy malnutrition contributes to skeletal muscle loss, immune dysfunction, and impaired tissue repair processes. In patients with heart failure, these alterations may aggravate the catabolic state associated with chronic cardiac dysfunction [46,47].

Hypoalbuminemia, a key component of the GNRI calculation, may also reflect systemic inflammation and impaired hepatic protein synthesis. Low albumin levels have repeatedly been associated with increased mortality and unfavorable clinical outcomes in cardiovascular populations [48,49]. The magnitude of the difference in serum albumin concentrations between groups was considerable, supporting the ability of GNRI to identify patients with substantially impaired nutritional status and greater clinical vulnerability.

In addition to mortality, patients with GNRI-defined nutritional risk required longer hospitalization, suggesting a higher burden of disease and a more complicated clinical course during admission. The median length of stay was approximately three days longer in patients with nutritional risk, highlighting the potential clinical and healthcare implications of impaired nutritional status in hospitalized patients with heart failure.

Recent studies suggest that the clinical significance of nutritional impairment in hospitalized older adults extends beyond conventional nutritional screening tools. In addition to anthropometric measures, several biochemical and hematological parameters, including serum albumin, hemoglobin, and inflammatory markers, have been associated with frailty, poor functional status, and adverse clinical outcomes in geriatric inpatients. Moreover, increasing evidence indicates that oxidative stress, adipokine imbalance, and chronic low-grade inflammation may contribute to the metabolic disturbances frequently observed in patients with impaired nutritional status. Together, these findings highlight that nutritional risk reflects not only reduced nutritional reserves but also broader alterations in metabolic and inflammatory pathways. In this context, GNRI may provide clinically relevant information regarding the overall health status and risk profile of hospitalized patients with cardiovascular disease [50,51].

The Kaplan–Meier analysis demonstrated only borderline statistical significance, whereas the association between lower GNRI values and in-hospital mortality remained significant after multivariable adjustment. This finding suggests that differences in baseline patient characteristics may have influenced the unadjusted survival analysis. After accounting for these factors, GNRI retained its prognostic value, supporting its role as a marker of adverse in-hospital outcomes independent of the clinical variables included in the model.

### 4.4. Nutritional Status and Cardiovascular Comorbidities

Subgroup analyses in the present study demonstrated that the association between GNRI and diabetes remained consistent across multiple clinical subgroups, including sex, age categories, smoking status, and the presence of cardiovascular comorbidities. These findings are broadly consistent with previous studies showing that the impact of nutritional impairment extends across diverse cardiovascular populations.

The relationship between nutritional status and cardiovascular disease has been extensively documented in the literature. Studies investigating patients with acute myocardial infarction have shown that impaired nutritional status is associated with poorer ventricular function and an increased risk of recurrent cardiovascular events [39,40]. These findings support the concept that nutritional impairment represents part of a broader syndrome of systemic vulnerability involving inflammation, metabolic dysregulation, and reduced physiological reserve [52]. The subgroup analyses should be interpreted cautiously because multiple comparisons were performed. Although the association between GNRI and diabetes mellitus appeared more pronounced among patients with abdominal obesity, only the interaction test for abdominal obesity reached statistical significance, whereas no significant interactions were observed for the other predefined subgroup variables. Therefore, these findings should be considered exploratory and hypothesis-generating rather than confirmatory. Given the known relationship between abdominal obesity, insulin resistance, and systemic inflammation, it is biologically plausible that body composition may influence the association between GNRI and diabetes mellitus [52]. However, this observation requires confirmation in larger prospective studies.

### 4.5. Clinical Implications

The potential clinical implications of these findings deserve consideration. The high prevalence of GNRI-defined nutritional risk observed in our cohort suggests that nutritional assessment may provide useful complementary information in patients hospitalized with heart failure. Because GNRI is based on routinely available parameters, including serum albumin, body weight, and height, it can be easily incorporated into clinical evaluation without additional costs or specialized testing.

Our findings also suggest that patients with GNRI-defined nutritional risk represent a clinically vulnerable subgroup characterized by a higher prevalence of diabetes mellitus, longer hospital stays, and increased in-hospital mortality. This finding suggests that GNRI may function, at least in part, as a broader marker of disease severity and physiological vulnerability rather than as an isolated nutritional predictor. Early recognition of nutritional risk may help identify patients who require closer clinical monitoring and a more comprehensive multidisciplinary approach during hospitalization. Previous studies have also suggested that nutritional interventions may contribute to improved outcomes in selected cardiovascular populations [52].

However, the clinical applicability of GNRI should be interpreted with caution. Although GNRI was associated with diabetes mellitus and in-hospital mortality in the present cohort, this study was not designed to determine whether GNRI provides incremental prognostic value beyond established heart failure markers such as NT-proBNP, left ventricular ejection fraction, NYHA functional class, or existing risk prediction models. Moreover, our findings do not demonstrate that the routine use of GNRI would alter clinical management or improve patient outcomes. Therefore, the present findings should be viewed as supporting the potential role of GNRI as an adjunctive marker of nutritional and clinical vulnerability rather than as evidence for its routine incorporation into risk stratification algorithms. These findings should be considered hypothesis-generating and require confirmation in larger prospective studies designed to determine whether adding GNRI to existing risk assessment strategies improves prognostic accuracy, influences clinical decision-making, and ultimately affects patient outcomes.

### 4.6. Limitations

Several limitations of this study should be acknowledged. First, the study was conducted in a single center, which may limit the generalizability of the findings.

Second, the observational design does not allow causal relationships between nutritional status and clinical outcomes to be established.

Third, GNRI is a screening tool and does not represent a comprehensive nutritional assessment, as it does not capture all dimensions of nutritional status, such as body composition, sarcopenia, or micronutrient deficiencies. Also, specific assessment of cardiac cachexia or sarcopenia was not performed.

Another limitation is the absence of an a priori sample size calculation. The study population was determined by the number of consecutively hospitalized patients meeting the eligibility criteria during the predefined recruitment period. Therefore, the possibility of limited statistical power for some subgroup and multivariable analyses cannot be excluded.

An additional limitation relates to the use of serum albumin as a component of the GNRI. Although albumin is widely used in nutritional risk assessment, its concentration may be influenced by factors unrelated to nutritional status, including systemic inflammation, volume overload, hepatic dysfunction, and dilutional effects. This issue may be particularly relevant in patients hospitalized with acute decompensated heart failure, in whom congestion and inflammatory activation are common. Consequently, GNRI should be interpreted as a nutritional risk indicator rather than a direct measure of nutritional status.

Several limitations should be considered when interpreting the present findings. First, the relatively small number of in-hospital deaths compared with the number of variables included in the fully adjusted models may have increased the risk of model overfitting. Although the observed associations remained broadly consistent across the different adjustment models, the fully adjusted results should be interpreted with caution and ideally confirmed in larger studies with a greater number of outcome events.

An additional limitation relates to the use of survival analysis for in-hospital mortality. Patients discharged alive were treated as censored observations; however, hospital discharge may represent informative censoring because it is closely related to clinical recovery and prognosis. Consequently, the results of the Kaplan–Meier and Cox regression analyses should be interpreted with caution.

Although several markers of heart failure severity, including left ventricular ejection fraction, renal function, anemia, and selected laboratory abnormalities, differed between groups, the number of in-hospital deaths limited the number of covariates that could be included in multivariable models without increasing the risk of overfitting. Consequently, residual confounding related to disease severity cannot be completely excluded, and the observed associations should be interpreted with appropriate caution.

Importantly, the association between GNRI and in-hospital mortality lost statistical significance after additional adjustment for markers of heart failure severity, including left ventricular ejection fraction, New York Heart Association functional class, ln(NT-proBNP), and estimated glomerular filtration rate. Therefore, although lower GNRI values identified patients with greater short-term clinical vulnerability, our findings do not support a definitive independent prognostic role of GNRI beyond established indicators of heart failure severity.

Finally, the present study focused primarily on in-hospital outcomes. Future studies including longer follow-up periods are needed to better understand the long-term prognostic implications of nutritional status in patients with heart failure.

## 5. Conclusions

In conclusion, lower GNRI values were associated with a higher prevalence of diabetes mellitus and identified patients with greater nutritional and clinical vulnerability during hospitalization for heart failure. Although lower GNRI values were associated with higher in-hospital mortality in the main models, this association was attenuated after adjustment for markers of heart failure severity. Therefore, GNRI should be interpreted as an adjunctive marker of nutritional and overall clinical vulnerability rather than as a definitive independent prognostic marker. Further prospective studies are needed to determine whether GNRI provides incremental clinical value beyond established heart failure risk markers.

## Figures and Tables

**Table 1:** Demographic, clinical, anthropometric, and laboratory characteristics of patients according to nutritional status assessed by GNRI.

Variable (Unit)	Category	All Patients (n = 278)	No Nutritional Risk Group, GNRI ≥ 98 (n = 161)	Nutritional Risk Group, GNRI < 98 (n = 117)	*p*-Value
Demographic and hospitalization characteristics					
Gender, n (%)	Male	132 (47.5%)	77 (47.8%)	55 (47.0%)	0.99
	Female	146 (52.5%)	84 (52.2%)	62 (53.0%)	
Age (years), Median (IQR)		74.00 (66.00–81.00)	74.00 (65.00–81.00)	74.00 (67.00–82.00)	0.162
Age group, n (%)	<65 years	57 (20.5%)	37 (23.0%)	20 (17.1%)	0.294
	≥65 years	221 (79.5%)	124 (77.0%)	97 (82.9%)	
Length of hospital stay, days, Median (IQR)		9.00 (7.00–12.00)	8.00 (5.00–11.00)	11.00 (9.00–14.00)	<0.001
In-hospital mortality, n (%)	No	227 (81.7%)	144 (89.4%)	83 (70.9%)	<0.001
	Yes	51 (18.3%)	17 (10.6%)	34 (29.1%)	
Prior hospitalizations (any cause), Median (IQR)		2.00 (2.00–3.00)	2.00 (2.00–3.00)	3.00 (2.00–4.00)	0.016
Nutritional status characteristics					
Weight (kg), Median (IQR)		84.00 (72.00–98.00)	85.00 (74.00–99.00)	82.50 (67.75–97.25)	0.076
Body mass index (kg/m^2^), Median (IQR)		30.12 (25.71–33.95)	30.69 (26.73–34.13)	29.69 (24.82–33.23)	0.079
Ideal body weight (Lorentz)		60.25 (55.00–66.50)	60.00 (55.00–66.50)	60.50 (55.00–65.00)	0.499
Abdominal obesity, n (%)	No	60 (21.6%)	29 (18.0%)	31 (26.5%)	0.121
	Yes	218 (78.4%)	132 (82.0%)	86 (73.5%)	
GNRI score, Median (IQR)		102.75 (91.73–108.70)	107.96 (104.30–111.53)	91.20 (87.11–95.30)	<0.001
CONUT score, Median (IQR)		2.00 (1.00–3.00)	1.00 (1.00–2.00)	3.00 (2.00–5.00)	<0.001
CONUT score classification, n (%)	Moderate nutritional risk	33 (11.9%)	4 (2.5%)	29 (24.8%)	<0.001
	Severe nutritional risk	3 (1.1%)	1 (0.6%)	2 (1.7%)	
	Mild nutritional risk	133 (47.8%)	73 (45.3%)	60 (51.3%)	
	No nutritional risk	109 (39.2%)	83 (51.6%)	26 (22.2%)	
Smoking status, n (%)	No	156 (56.1%)	103 (64.0%)	53 (45.3%)	<0.001
	Yes	122 (43.9%)	58 (36.0%)	64 (54.7%)	
Alcohol consumption, n (%)	No	171 (61.5%)	128 (79.5%)	43 (36.8%)	<0.001
	Yes	107 (38.5%)	33 (20.5%)	74 (63.2%)	
Laboratory parameters					
Hemoglobin (g/dL), Median (IQR)		12.14 (10.60–13.50)	13.09 (11.80–14.35)	11.00 (9.21–12.22)	<0.001
Hematocrit (%), Median (IQR)		37.30 (30.59–41.40)	39.90 (36.43–43.26)	31.00 (23.70–37.50)	<0.001
Blood glucose (at admission), Median (IQR)		124.00 (106.00–165.00)	121.00 (104.00–157.00)	126.00 (108.00–167.25)	0.382
Serum iron (µmol/L), Median (IQR)		9.75 (5.40–13.57)	10.45 (6.70–14.50)	7.50 (4.70–13.00)	0.003
Ferritin (ng/mL), Median (IQR)		18.00 (13.90–26.70)	34.85 (19.38–51.02)	15.30 (11.90–17.90)	<0.001
Uric acid (µmol/L), Median (IQR)		339.50 (266.25–446.75)	371.50 (287.75–461.50)	306.50 (243.25–396.50)	<0.001
Creatinine at admission (mg/dL), Median (IQR)		1.04 (0.85–1.28)	1.13 (0.88–1.47)	0.95 (0.80–1.10)	<0.001
eGFR at admission (mL/min/1.73 m^2^), Median (IQR)		60.28 (42.79–77.81)	60.28 (41.26–77.01)	60.40 (45.59–78.55)	0.563
Albumin (g/dL), Median (IQR)		3.75 (3.33–4.32)	4.25 (3.95–4.50)	3.33 (3.07–3.60)	<0.001
Potassium at admission (mmol/L), Median (IQR)		4.20 (3.59–4.60)	4.40 (4.03–4.80)	3.58 (3.13–4.30)	<0.001
Sodium at admission (mmol/L), Median (IQR)		139.00 (136.00–142.00)	140.00 (138.00–143.00)	137.00 (131.00–140.00)	<0.001
NT-proBNP (pg/mL), Median (IQR)		4716.00 (2051.25–15,158.25)	4277.00 (2246.00–13,741.00)	5578.00 (1937.00–16,032.00)	0.598
AST (U/L), Median (IQR)		27.00 (19.00–49.50)	23.00 (17.00–32.00)	40.00 (24.00–62.00)	<0.001
ALT (U/L), Median (IQR)		23.00 (15.00–43.00)	22.00 (14.75–33.25)	32.00 (18.00–55.00)	<0.001

Note: Denominators may vary across variables because a limited number of clinical, echocardiographic, and treatment-related parameters were unavailable for all patients. Percentages were calculated using the number of available observations for each variable. Percentages and summary statistics were calculated using the number of available observations for each variable. Due to incomplete laboratory documentation, denominators varied for selected parameters, including ferritin (n = 137), creatinine (n = 277), eGFR (n = 277), NT-proBNP (n = 144), AST (n = 273), and ALT (n = 273).

**Table 2:** Cardiovascular characteristics, comorbidities, and clinical complications according to nutritional status assessed by GNRI.

Variable (Unit)	Category	All (n = 278)	No Nutritional Risk Group, GNRI ≥ 98 (n = 161)	Nutritional Risk Group, GNRI < 98 (n = 117)	*p*-Value
Cardiovascular status					
Left ventricular ejection fraction (%), Median (IQR)		40.00 (31.50–50.00)	45.00 (35.00–50.00)	35.00 (30.00–40.00)	<0.001
Atrial fibrillation, n (%)	No	135 (48.6%)	73 (45.3%)	62 (53.0%)	0.355
	Yes	47 (16.9%)	27 (16.8%)	20 (17.1%)	
Heart failure type, n (%)	Right	3 (1.1%)	3 (1.9%)	0 (0.0%)	0.032
	Global	180 (64.7%)	95 (59.0%)	85 (72.6%)	
	Left	95 (34.2%)	63 (39.1%)	32 (27.4%)	
NYHA class, n (%)	II	56 (20.1%)	36 (22.4%)	20 (17.1%)	0.522
	III	138 (49.6%)	79 (49.1%)	59 (50.4%)	
	IV	84 (30.2%)	46 (28.6%)	38 (32.5%)	
Angina pectoris, n (%)	No	160 (57.6%)	118 (73.3%)	42 (35.9%)	<0.001
	Yes	118 (42.4%)	43 (26.7%)	75 (64.1%)	
Coronary heart disease, n (%)	No	164 (59.0%)	132 (82.0%)	32 (27.4%)	<0.001
	Yes	114 (41.0%)	29 (18.0%)	85 (72.6%)	
Prior myocardial infarction, n (%)	No	182 (65.5%)	132 (82.0%)	50 (42.7%)	<0.001
	Yes	96 (34.5%)	29 (18.0%)	67 (57.3%)	
LVEF category, n (%)	Preserved (≥50%)	78 (37.0%)	57 (49.1%)	21 (22.1%)	<0.001
	Mildly reduced (40–49%)	72 (34.1%)	31 (26.7%)	41 (43.2%)	
	Reduced (<40%)	61 (28.9%)	28 (24.1%)	33 (34.7%)	
Conduction disorder (block), n (%)	No	141 (50.7%)	95 (59.0%)	46 (39.3%)	0.002
	Yes	137 (49.3%)	66 (41.0%)	71 (60.7%)	
Comorbidities and complications					
Diabetes mellitus, n (%)	No	110 (39.6%)	79 (49.1%)	31 (26.5%)	<0.001
	Yes	168 (60.4%)	82 (50.9%)	86 (73.5%)	
Anemia, n (%)	No	165 (59.4%)	110 (68.3%)	55 (47.0%)	<0.001
	Yes	113 (40.6%)	51 (31.7%)	62 (53.0%)	
Thrombotic/thromboembolic complications, n (%)	No	205 (73.7%)	132 (82.0%)	73 (62.4%)	<0.001
	Yes	73 (26.3%)	29 (18.0%)	44 (37.6%)	
Prior ischemic stroke, n (%)	No	233 (83.8%)	144 (89.4%)	89 (76.1%)	0.005
	Yes	45 (16.2%)	17 (10.6%)	28 (23.9%)	
Ischemic stroke (acute), n (%)	Yes	6 (2.2%)	3 (1.9%)	3 (2.6%)	0.699
	No	272 (97.8%)	158 (98.1%)	114 (97.4%)	
Diabetic neuropathy, n (%)	No	207 (74.5%)	145 (90.1%)	62 (53.0%)	<0.001
	Yes	71 (25.5%)	16 (9.9%)	55 (47.0%)	
Chronic kidney disease, n (%)	No	117 (42.1%)	73 (45.3%)	44 (37.6%)	0.243
	Yes	161 (57.9%)	88 (54.7%)	73 (62.4%)	

Note: Denominators may vary across variables because a limited number of clinical, echocardiographic, and treatment-related parameters were unavailable for all patients. Percentages were calculated using the number of available observations for each variable. Percentages were calculated using the number of available observations for each variable.

**Table 3:** Pharmacological treatment according to nutritional status assessed by GNRI.

Variable (Therapeutic Class)	Category	All (n = 278)	No Nutritional Risk Group, GNRI ≥ 98 (n = 161)	Nutritional Risk Group, GNRI < 98 (n = 117)	*p*-Value
ACE inhibitor therapy, n (%)	No	98 (35.3%)	51 (31.7%)	47 (40.2%)	0.251
	Yes	175 (62.9%)	105 (65.2%)	70 (59.8%)	
ARB therapy, n (%)	No	182 (65.5%)	103 (64.0%)	79 (67.5%)	0.627
	Yes	96 (34.5%)	58 (36.0%)	38 (32.5%)	
ARNI therapy (sacubitril/valsartan), n (%)	No	266 (95.7%)	152 (94.4%)	114 (97.4%)	1.000
	Yes	7 (2.5%)	4 (2.5%)	3 (2.6%)	
Beta-blocker therapy, n (%)	No	39 (14.0%)	23 (14.3%)	16 (13.7%)	0.94
	Yes	234 (84.2%)	133 (82.6%)	101 (86.3%)	
Anticoagulant therapy, n (%)	No	143 (51.4%)	80 (49.7%)	63 (53.8%)	0.573
	Yes	135 (48.6%)	81 (50.3%)	54 (46.2%)	
NOAC therapy, n (%)	No	226 (81.3%)	129 (80.1%)	97 (82.9%)	0.666
	Yes	52 (18.7%)	32 (19.9%)	20 (17.1%)	
Oral antidiabetic therapy, n (%)	No	177 (63.7%)	110 (68.3%)	67 (57.3%)	0.077
	Yes	101 (36.3%)	51 (31.7%)	50 (42.7%)	
Insulin therapy, n (%)	No	208 (74.8%)	129 (80.1%)	79 (67.5%)	0.024
	Yes	70 (25.2%)	32 (19.9%)	38 (32.5%)	
Mineralocorticoid receptor antagonist therapy, n (%)	No	91 (32.7%)	52 (32.3%)	39 (33.3%)	0.990
	Yes	187 (67.3%)	109 (67.7%)	78 (66.7%)	
Antiplatelet therapy, n (%)	No	165 (59.4%)	96 (59.6%)	69 (59.0%)	0.990
	Yes	113 (40.6%)	65 (40.4%)	48 (41.0%)	

**Table 4:** Association between GNRI and diabetes in patients with heart failure.

Variable	Model 1 OR (95% CI)	*p*	Model 2 OR (95% CI)	*p*	Model 3 OR (95% CI)	*p*-Value
GNRI (continuous)	0.936 (0.914–0.959)	<0.001	0.930 (0.906–0.954)	<0.001	0.890 (0.860–0.921)	<0.001
GNRI quartiles						
Q4 (highest GNRI)	Reference		Reference		Reference	
Q3	1.41 (0.85–2.34)	0.180	1.38 (0.82–2.30)	0.210	1.32 (0.77–2.25)	0.300
Q2	1.92 (1.13–3.26)	0.016	1.86 (1.08–3.21)	0.025	1.74 (1.00–3.02)	0.048
Q1 (lowest GNRI)	2.58 (1.48–4.48)	<0.001	2.43 (1.37–4.31)	0.002	2.21 (1.23–3.96)	0.008
*p* for trend	<0.001		0.002		0.006	
GNRI-defined nutritional risk	2.11 (1.31–3.40)	0.002	1.96 (1.20–3.21)	0.007	1.88 (1.14–3.08)	0.014

Legend: OR = odds ratio; CI = confidence interval. Model 1: unadjusted. Model 2: adjusted for age and sex. Model 3: additionally adjusted for body mass index, abdominal obesity, smoking status, alcohol consumption, coronary artery disease, angina pectoris, previous myocardial infarction, ischemic stroke, and hypertension.

**Table 5:** Association between GNRI and mortality in patients with heart failure.

Variable	Model 1 HR (95% CI)	*p*-Value	Model 2 HR (95% CI)	*p*-Value	Model 3 HR (95% CI)	*p*-Value	Model 4 HR (95% CI)	*p*-Value
GNRI (continuous)	0.964 (0.931–0.998)	0.039	0.960 (0.925–0.996)	0.031	0.957 (0.920–0.996)	0.031	0.957 (0.920–0.996)	0.031
GNRI quartiles								
Q4 (highest GNRI)	Reference		Reference		Reference		Reference	
Q3	1.18 (0.55–2.54)	0.667	1.22 (0.56–2.64)	0.621	1.29 (0.59–2.82)	0.523	1.31 (0.60–2.88)	0.497
Q2	1.67 (0.78–3.55)	0.189	1.74 (0.81–3.74)	0.155	1.86 (0.85–4.06)	0.118	1.92 (0.88–4.18)	0.101
Q1 (lowest GNRI)	2.36 (1.12–4.98)	0.024	2.45 (1.15–5.23)	0.020	2.61 (1.21–5.63)	0.014	2.72 (1.25–5.92)	0.011
*p* for trend	0.021		0.017		0.012		0.009	
GNRI-defined nutritional risk	2.12 (1.03–4.36)	0.041	2.25 (1.08–4.69)	0.030	2.38 (1.12–5.03)	0.024	2.41 (1.15–5.05)	0.019

Legend: HR = hazard ratio; CI = confidence interval. Model 1: unadjusted. Model 2: adjusted for age and sex. Model 3: additionally adjusted for BMI, abdominal obesity, smoking, and alcohol consumption. Model 4: further adjusted for coronary heart disease, angina pectoris, prior myocardial infarction, prior stroke, and hypertension.

**Table 6:** Subgroup analyses of the association between GNRI and diabetes mellitus in patients with heart failure.

Subgroup	GNRI (Continuous) OR (95% CI)	*p*-Value	GNRI-Defined Nutritional Risk OR (95% CI)	*p*-Value	*p* for Interaction
Gender					0.31
Male	0.918 (0.892–0.956)	<0.001	2.24 (1.31–3.40)	0.002	
Female	0.956 (0.917–0.983)	0.003	1.73 (1.14–3.08)	0.014	
Age					0.18
≥70 years	0.955 (0.929–0.982)	0.001	1.96 (1.23–3.12)	0.005	
<70 years	0.865 (0.811–0.924)	<0.001	2.45 (1.41–4.25)	0.002	
BMI ≥ 30 kg/m^2^					0.22
Yes	0.912 (0.878–0.947)	<0.001	2.37 (1.41–3.99)	0.001	
No	0.947 (0.917–0.978)	<0.001	1.81 (1.12–2.95)	0.016	
Abdominal obesity					0.04
Yes	0.905 (0.853–0.920)	<0.001	2.72 (1.61–4.60)	<0.001	
No	0.995 (0.954–1.038)	0.809	1.08 (0.52–2.25)	0.84	
Smoking					0.27
Yes	0.919 (0.883–0.956)	<0.001	2.31 (1.21–3.61)	0.008	
No	0.944 (0.914–0.975)	<0.001	1.81 (1.19–3.09)	0.007	
Alcohol use					0.41
Yes	0.931 (0.896–0.968)	<0.001	2.01 (1.18–3.42)	0.010	
No	0.938 (0.908–0.969)	<0.001	1.95 (1.21–3.13)	0.006	
Atrial fibrillation (AF)					0.36
Yes	0.927 (0.900–0.968)	<0.001	2.17 (1.19–3.57)	0.010	
No	0.947 (0.908–0.971)	<0.001	1.76 (1.17–3.04)	0.009	
Angina pectoris					0.44
Yes	0.928 (0.892–0.965)	<0.001	2.12 (1.19–3.77)	0.011	
No	0.941 (0.911–0.973)	<0.001	1.91 (1.18–3.10)	0.009	
Coronary heart disease					0.33
Yes	0.922 (0.899–0.965)	<0.001	2.26 (1.25–3.32)	0.004	
No	0.951 (0.915–0.977)	<0.001	1.71 (1.14–3.09)	0.014	
Prior myocardial infarction (MI)					0.29
Yes	0.926 (0.891–0.963)	<0.001	2.18 (1.26–3.78)	0.005	
No	0.944 (0.914–0.975)	<0.001	1.90 (1.18–3.05)	0.008	
Stroke					0.37
Yes	0.932 (0.895–0.970)	<0.001	2.09 (1.19–3.67)	0.010	
No	0.940 (0.910–0.971)	<0.001	1.92 (1.18–3.12)	0.009	
Hypertension					0.48
Yes	0.937 (0.907–0.968)	<0.001	1.98 (1.23–3.20)	0.005	
No	0.945 (0.904–0.987)	0.011	1.77 (0.98–3.21)	0.059	
Anemia					0.25
Yes	0.921 (0.887–0.956)	<0.001	2.28 (1.34–3.86)	0.002	
No	0.948 (0.918–0.979)	0.001	1.83 (1.13–2.97)	0.014	
Dyslipidemia					0.39
Yes	0.926 (0.902–0.965)	<0.001	2.04 (1.27–3.28)	0.003	
No	0.953 (0.917–0.989)	0.004	1.89 (1.10–3.24)	0.021	

Legend: OR = odds ratio; CI = confidence interval; *p* for interaction was obtained by adding an interaction term between GNRI and each subgroup variable in the logistic regression model.

## Data Availability

The data presented in this study are available on reasonable request from the corresponding author. The data are not publicly available due to ethical and privacy restrictions related to patient confidentiality.

## Supplementary Materials

The following supporting information can be downloaded at: https://www.mdpi.com/article/10.3390/nu18132198/s1, Table S1. Extended laboratory characteristics according to GNRI-defined nutritional risk; Table S2. Additional cardiovascular comorbidities and clinical complications according to GNRI-defined nutritional status; Table S3. Pharmacological treatment characteristics according to GNRI-defined nutritional status; Table S4. Multivariable logistic regression analysis of in-hospital mortality; Table S5. Sensitivity Cox regression analysis using a parsimonious adjustment model; Table S6. Sensitivity Cox proportional hazards analysis adjusted for markers of heart failure severity.

## Abbreviations

The following abbreviations are used in this manuscript:

ALT	Alanine Aminotransferase
AST	Aspartate Aminotransferase
BMI	Body Mass Index
CAD	Coronary Artery Disease
CI	Confidence Interval
CONUT	Controlling Nutritional Status
eGFR	Estimated Glomerular Filtration Rate
ESC	European Society of Cardiology
GNRI	Geriatric Nutritional Risk Index
HF	Heart Failure
HFmrEF	Heart Failure with Mildly Reduced Ejection Fraction
HFpEF	Heart Failure with Preserved Ejection Fraction
HFrEF	Heart Failure with Reduced Ejection Fraction
HR	Hazard Ratio
ICU	Intensive Care Unit
IQR	Interquartile Range
LVEF	Left Ventricular Ejection Fraction
MI	Myocardial Infarction
NT-proBNP	N-terminal Pro-B-type Natriuretic Peptide
NYHA	New York Heart Association
OR	Odds Ratio
SD	Standard Deviation
SPSS	Statistical Package for the Social Sciences
