# Supplementary material for: Reduced Geriatric Nutritional Risk Index Is Associated with Prevalent Diabetes Mellitus and In-Hospital Mortality in Patients Hospitalized with Heart Failure
Source: Nutrients. 2026 Jul 7;18(13):2198. doi: 10.3390/nu18132198 (PMC13364032; doi:10.3390/nu18132198)
Supplement: Supplementary file 1 [file nutrients-18-02198-s001.zip › nutrients-4383002-supplementary.pdf]

Table S1. Extended laboratory characteristics according to GNRI-defined nutritional risk

Variable (unit)	Category	All patients (n=278)	No nutritional risk group, GNRI ≥98 (n=161)	Nutritional risk group, GNRI <98 (=117)	p-value
**Demographic and hospitalization characteristics**					
Residence, n(%)	Urban	144 (51.8%)	84 (52.2%)	60 (51.3%)	0.98
	Rural	134 (48.2%)	77 (47.8%)	57 (48.7%)	
Type of hospitalization, n(%)	Emergency	94 (33.8%)	57 (35.4%)	37 (31.6%)	0.597
	Elective	184 (66.2%)	104 (64.6%)	80 (68.4%)	
**Nutritional status characteristics**					
Height (cm), Median(IQR)		167.00 (160.00–173.00)	167.00 (160.00–173.00)	167.00 (160.00–174.00)	0.385
Ideal body weight (Lorentz)		60.25 (55.00–66.50)	60.00 (55.00–66.50)	60.50 (55.00–65.00)	0.499
Weight/IBW ratio, Median(IQR)		1.38 (1.17–1.54)	1.39 (1.22–1.56)	1.36 (1.10–1.52)	0.052
**Laboratory parameters**					
Leukocytes ($\times10^9$/L), Median(IQR)		8.60 (6.69–11.09)	8.38 (6.56–10.34)	8.94 (6.84–11.55)	0.106
Lymphocytes ($\times10^9$/L), Median(IQR)		1.91 ± 0.58	1.91 ± 0.60	1.90 ± 0.55	0.858
Erythrocytes ($\times10^{12}$/L), Median(IQR)		4.39 (3.90–4.80)	4.46 (4.01–4.86)	4.26 (3.76–4.69)	0.006
Platelets ($\times10^9$/L), Median(IQR)		210.65 (173.25–271.70)	208.40 (175.80–266.70)	221.00 (169.00–282.00)	0.703
MCV (fL), Median(IQR)		88.50 (85.09–92.63)	89.20 (85.41–92.97)	87.60 (83.91–92.39)	0.142
MCH (pg), Median(IQR)		29.40 (27.73–30.70)	29.60 (27.81–30.70)	29.10 (27.65–30.64)	0.27
MCHC (g/dL), Median(IQR)		32.80 (31.90–33.80)	32.60 (31.87–33.60)	33.10 (32.00–34.00)	0.149
Direct bilirubin (mg/dL)		0.34 (0.24–0.50)	0.36 (0.24–0.50)	0.33 (0.24–0.51)	0.84
Total bilirubin (mg/dL), Median(IQR)		0.70 (0.50–1.01)	0.70 (0.50–1.09)	0.69 (0.50–1.00)	0.438
Total cholesterol (mmol/L), Median(IQR)		3.95 (3.20–5.08)	4.17 (3.25–5.12)	3.78 (3.02–4.85)	0.086
HDL cholesterol (mmol/L), Median(IQR)		1.10 (0.80–1.32)	1.09 (0.86–1.33)	1.10 (0.76–1.29)	0.327
Triglycerides (mmol/L), Median(IQR)		1.31 (0.93–1.81)	1.33 (0.96–1.86)	1.28 (0.91–1.75)	0.302

Table S2. Additional cardiovascular characteristics, clinical complications and associated conditions

Variable (unit)	Category	All (n=278)	No nutritional risk group, GNRI ≥98 (n=161)	Nutritional risk group, GNRI <98 (=117)	p-value
**Cardiovascular status**					

QRS complex, Median(IQR)		102.00 (90.00–120.00)	104.00 (90.00–120.00)	100.00 (90.00–122.00)	0.834
QTc duration (ms), Median(IQR)		437.00 (413.00–463.00)	436.00 (412.00–458.00)	442.50 (416.25–469.50)	0.053
Systolic blood pressure (mmHg), Median(IQR)		140.00 (120.00–160.00)	140.00 (120.00–160.00)	140.00 (120.00–160.00)	0.926
Diastolic blood pressure (mmHg), Median(IQR)		80.00 (70.00–90.00)	80.00 (70.00–90.00)	80.00 (70.00–90.00)	0.643
Atrial fibrillation type, n(%)	No	135 (48.6%)	73 (45.3%)	62 (53.0%)	0.355
	Paroxysmal	47 (16.9%)	27 (16.8%)	20 (17.1%)	
	Permanent	96 (34.5%)	61 (37.9%)	35 (29.9%)	
Heart rate >100 bpm, n(%)	No	189 (68.0%)	102 (63.4%)	87 (74.4%)	0.145
	Yes	84 (30.2%)	54 (33.5%)	30 (25.6%)	
Prior heart failure diagnosis, n(%)	No	106 (38.1%)	61 (37.9%)	45 (38.5%)	0,99
	Yes	172 (61.9%)	100 (62.1%)	72 (61.5%)	
**Comorbidities and complications**					
Dyslipidemia, n(%)	No	111 (39.9%)	71 (44.1%)	40 (34.2%)	0.123
	Yes	167 (60.1%)	90 (55.9%)	77 (65.8%)	
Hyperuricemia, n(%)	No	178 (64.0%)	110 (68.3%)	68 (58.1%)	0.104
	Yes	100 (36.0%)	51 (31.7%)	49 (41.9%)	
Acid–base disorders, n(%)	No	243 (87.4%)	158 (98.1%)	85 (72.6%)	**<0.001**
	Yes	35 (12.6%)	3 (1.9%)	32 (27.4%)	
Edema, n(%)	No	155 (55.8%)	99 (61.5%)	56 (47.9%)	**0.033**
	Yes	123 (44.2%)	62 (38.5%)	61 (52.1%)	
Psychiatric illness, n(%)	No	208 (74.8%)	130 (80.7%)	78 (66.7%)	**0.011**
	Yes	70 (25.2%)	31 (19.3%)	39 (33.3%)	
	No	272 (97.8%)	158 (98.1%)	114 (97.4%)	
Fibrosis, n(%)	Yes	41 (14.7%)	20 (12.4%)	21 (17.9%)	0.266
	No	237 (85.3%)	141 (87.6%)	96 (82.1%)	
Pneumonia, n(%)	Yes	44 (15.8%)	18 (11.2%)	26 (22.2%)	**0.02**
	No	234 (84.2%)	143 (88.8%)	91 (77.8%)	
Bronchopneumonia, n(%)	Yes	30 (10.8%)	14 (8.7%)	16 (13.7%)	0.261
	No	248 (89.2%)	147 (91.3%)	101 (86.3%)	
Urinary tract infection, n(%)	No	169 (60.8%)	118 (73.3%)	51 (43.6%)	**<0.001**
	Yes	109 (39.2%)	43 (26.7%)	66 (56.4%)	
Hyperglycemia (at admission), n(%)	No	96 (34.5%)	57 (35.4%)	39 (33.3%)	0.818
	Yes	182 (65.5%)	104 (64.6%)	78 (66.7%)	

Table S3. Additional Pharmacological Therapies According to GNRI-Defined Nutritional Risk

Variable (Therapeutic class)	Category	All (N=278)	No nutritional risk group, GNRI ≥98 (n=161)	Nutritional risk group, GNRI <98 (=117)	p-value
Antibiotic therapy, n(%)	No	124 (44.6%)	66 (41.0%)	58 (49.6%)	0.396
	Yes	72 (25.9%)	33 (20.5%)	39 (33.3%)	
Central antihypertensive therapy, n(%)	No	261 (93.9%)	149 (92.5%)	112 (95.7%)	0.402
	Yes	17 (6.1%)	12 (7.5%)	5 (4.3%)	
Heart rate-lowering therapy (ivabradine) , n(%)	No	272 (97.8%)	158 (98.1%)	114 (97.4%)	0.699
	Yes	6 (2.2%)	3 (1.9%)	3 (2.6%)	
Loop diuretic therapy, n(%)	No	43 (15.5%)	25 (15.5%)	18 (15.4%)	1.000
	Yes	230 (82.7%)	131 (81.4%)	99 (84.6%)	
Thiazide/thiazide-like diuretic therapy, n(%)	No	194 (69.8%)	113 (70.2%)	81 (69.2%)	0.969
	Yes	84 (30.2%)	48 (29.8%)	36 (30.8%)	
Cardiac glycoside therapy, n(%)	No	234 (84.2%)	132 (82.0%)	102 (87.2%)	0.315
	Yes	44 (15.8%)	29 (18.0%)	15 (12.8%)	
Statin therapy, n(%)	No	138 (49.6%)	79 (49.1%)	59 (50.4%)	0.919
	Yes	140 (50.4%)	82 (50.9%)	58 (49.6%)	
Fibrate therapy, n(%)	No	262 (94.2%)	154 (95.7%)	108 (92.3%)	0.357
	Yes	16 (5.8%)	7 (4.3%)	9 (7.7%)	
Calcium channel blocker therapy, n(%)	No	180 (64.7%)	97 (60.2%)	83 (70.9%)	0.086
	Yes	98 (35.3%)	64 (39.8%)	34 (29.1%)	

Table S4. Multivariable logistic regression analysis for in-hospital mortality

Variable	OR (95% CI)	p-value
GNRI (continuous)	0.928 (0.895–0.962)	<0.001
Age	0.983 (0.953–1.014)	0.289
Sex	0.687 (0.354–1.331)	0.265
Body mass index (BMI)	1.002 (0.949–1.059)	0.934
Diabetes mellitus	0.401 (0.184–0.875)	0.022
Coronary heart disease	0.996 (0.486–2.041)	0.991

Legend: OR = odds ratio; CI = confidence interval. Multivariable logistic regression model adjusted for age, sex, body mass index, diabetes mellitus, and coronary heart disease.

Table S5. Sensitivity analyses using parsimonious Cox regression models for in-hospital mortality

Variable	Model 1 HR (95% CI)	p-value	Model 2 HR (95% CI)	p-value	Model 3 HR (95% CI)	p-value	Model 4 HR (95% CI)	p-value
GNRI (continuous)	0.975 (0.950–1.001)	0.063	0.974 (0.948–1.000)	0.052	0.972 (0.945–1.000)	0.049	0.975 (0.947–1.004)	0.088

Legend: HR = hazard ratio; CI = confidence interval. Model 1: unadjusted. Model 2: adjusted for age and sex. Model 3: adjusted for age, sex, diabetes mellitus, and coronary heart disease. Model 4: adjusted for age, sex, body mass index, diabetes mellitus, and coronary heart disease.

Table S6. Sensitivity Cox proportional hazards analysis adjusted for markers of heart failure severity

Variable	HR	95% CI	p-value
GNRI	1.014	0.972–1.057	0.529
LVEF (%)	1.011	0.963–1.061	0.660
NYHA functional class	0.887	0.372–2.115	0.786
ln(NT-proBNP)	2.364	1.306–4.280	0.004
eGFR (mL/min/1.73 m^2)	1.023	1.005–1.041	0.011

Legend: HR = hazard ratio; CI = confidence interval. Cox proportional hazards model adjusted for markers of heart failure severity. Adjusted for left ventricular ejection fraction (LVEF), New York Heart Association (NYHA) functional class, ln(NT-proBNP), and estimated glomerular filtration rate (eGFR). NT-proBNP values were natural log-transformed because of their markedly right-skewed distribution.
